# Possible Factors Influencing the Seroprevalence of Dengue among Residents of the Forest Fringe Areas of Peninsular Malaysia

**DOI:** 10.1155/2020/1019238

**Published:** 2020-05-25

**Authors:** Juraina Abd-Jamil, Romano Ngui, Syahrul Nellis, Rosmadi Fauzi, Ai Lian Yvonne Lim, Karuthan Chinna, Chee-Sieng Khor, Sazaly AbuBakar

**Affiliations:** ^1^Tropical Infectious Diseases Research and Education Centre (TIDREC), University Malaya, Kuala Lumpur 50603, Malaysia; ^2^Department of Parasitology, Faculty of Medicine, University Malaya, Kuala Lumpur 50603, Malaysia; ^3^Department of Geography, Faculty of Arts and Social Sciences, University Malaya, Kuala Lumpur 50603, Malaysia; ^4^Department of Social Preventive Medicine, University Malaya, Kuala Lumpur 50603, Malaysia; ^5^Faculty of Health and Medical Sciences, Taylor's University Lakeside Campus, No. 1, Jalan Taylor's, Subang Jaya 47500, Selangor, Malaysia; ^6^Department of Medical Microbiology, University Malaya, Kuala Lumpur 50603, Malaysia

## Abstract

Dengue is an endemic mosquito-borne viral disease prevalent in many urban areas of the tropic, especially the Southeast Asia. Its presence among the indigenous population of Peninsular Malaysia (*Orang Asli*), however, has not been well described. The present study was performed to investigate the seroprevalence of dengue among the *Orang Asli* (OA) residing at the forest fringe areas of Peninsular Malaysia and determine the factors that could affect the transmission of dengue among the OA. Eight OA communities consisting of 491 individuals were recruited. From the study, at least 17% of the recruited study participants were positive for dengue IgG, indicating past exposure to dengue. Analysis on the demographic and socioeconomic variables suggested that high seroprevalence of dengue was significantly associated with those above 13 years old and a low household income of less than MYR500 (USD150). It was also associated with the vast presence of residential areas and the presence of a lake. Remote sensing analysis showed that higher land surface temperatures and lower land elevations also contributed to higher dengue seroprevalence. The present study suggested that both demographic and geographical factors contributed to the increasing risk of contracting dengue among the OA living at the forest fringe areas of Peninsular Malaysia. The OA, hence, remained vulnerable to dengue.

## 1. Introduction

Dengue is a mosquito-borne viral disease that causes an estimated 390 million infections annually of which 96 million resulted in clinical manifestations [1]. The disease is caused by dengue virus (DENV), which is transmitted by the *Aedes* sp. mosquitoes. There are four dengue virus serotypes: dengue type 1 virus (DENV-1), dengue type 2 virus (DENV-2), dengue type 3 virus (DENV-3), and dengue type 4 virus (DENV-4). All four DENV serotypes circulate in most of the dengue-endemic regions such as in Indonesia, Vietnam, Thailand, and Malaysia. Once infected with the virus, dengue may manifest as clinically unapparent or asymptomatic infection, undifferentiated fever, or as severe dengue.

Dengue was thought to have originated from the sylvatic cycle where the virus circulated among nonhuman primates and the tree top-dwelling *Aedes* sp. mosquitoes such as *Aedes niveus* and *A. luteocephalus* [2]. At an estimated 1,000 years ago, dengue spilled into the human populations [3] and became endemic following rapid, unplanned urbanization and massive population migration from the rural to the urban areas [4]. In the endemic human cycle, dengue is transmitted mainly by the vectors *A. aegypti* and *A. albopictus* [4, 5]. The vectors are widely found in the subtropical and tropical regions of the world. *A. albopictus* has been suggested to bridge the sylvatic and urban cycle of dengue due to their abundance in the rural and forested areas in comparison to *A. aegypti* [6, 7].

Malaysia is among the earlier countries that reported dengue hyperendemicity and dengue hemorrhagic fever [8]. The dengue surveillance system implemented in Malaysia operates by receiving notifications of febrile dengue cases from both the government and private hospitals and clinics. The system, however, did not wholly include the underserved and economically marginalized communities such as the indigenous people of Peninsular Malaysia locally known as the *Orang Asli* (OA), as most still seek medical advice from the village shamans and use traditional medicines for treatments [9]. Earlier reports on dengue prevalence among the forest fringe populations were published in 1956 and 1958 [10, 11], which reported that virtually all adults from the rural communities of ethnic Malays had been exposed to dengue [10]. The study conducted two years later in 1958 showed that about 90% of the rural ethnic Malays and the OA in Bukit Lanong and Cameron Highlands, Pahang, had neutralizing antibodies against DENV [11]. These two studies predated the development of more accurate dengue serological assays. Results obtained from these earlier studies, hence, could be reflective of an imperfect laboratory tool where the ELISA used could highly cross-reacted with other arboviruses. Another study conducted 30 years later, however, showed a similar dengue seroprevalence (80%) among the forest fringe populations in Malaysia [12]. Nevertheless, more recent studies demonstrated that a wide difference of dengue seroprevalence existed between the rural populations in East Malaysia (24%) [13] and Peninsular Malaysia (91%) [14]. These studies suggested that dengue transmission and prevalence varied over time for populations residing in the rural and forest fringe areas of Malaysia. Many factors could have contributed to the differing dengue prevalence in these populations. The present study attempted to determine these factors by investigating the potential influence that demographic and socioeconomic variables as well as land cover and physical environmental factors might have on dengue IgG seroprevalence. The serosurvey, land cover, and remote sensing analysis were performed in eight different OA villages distributed across the states in Peninsular Malaysia. This represents a cross-sectional study using convenience-sampling method among voluntary members of different OA villages.

## 2. Methods

### 2.1. Ethics Approval and Consent to Participate

This study was approved by the Ethics Committee of the University Malaya Medical Centre (UMMC; MEC Ref. 824.11) and the Department of Orang Asli Development or locally known as the Jabatan Kemajuan Orang Asli (JAKOA), Ministry of Rural and Regional Development Malaysia.

Prior to obtaining informed consent, members of the community were given a briefing on the study. Participants who agreed to participate provided an oral consent to the trained field assistants, followed by a written consent. In instances where oral consent was received, but written consent could not be obtained due to illiteracy, the participants would provide either a thumbprint (for participants older than 13 years old) or written and oral consent from the legal guardian. The UMMC MEC 824.11 approval permitted both the use of thumbprints and a legal guardian's signature as indicators of written consent. Study participation was voluntary, and participants could withdraw at any time during study duration by informing the study coordinator.

### 2.2. Study Population and Area

**Figure 1:**
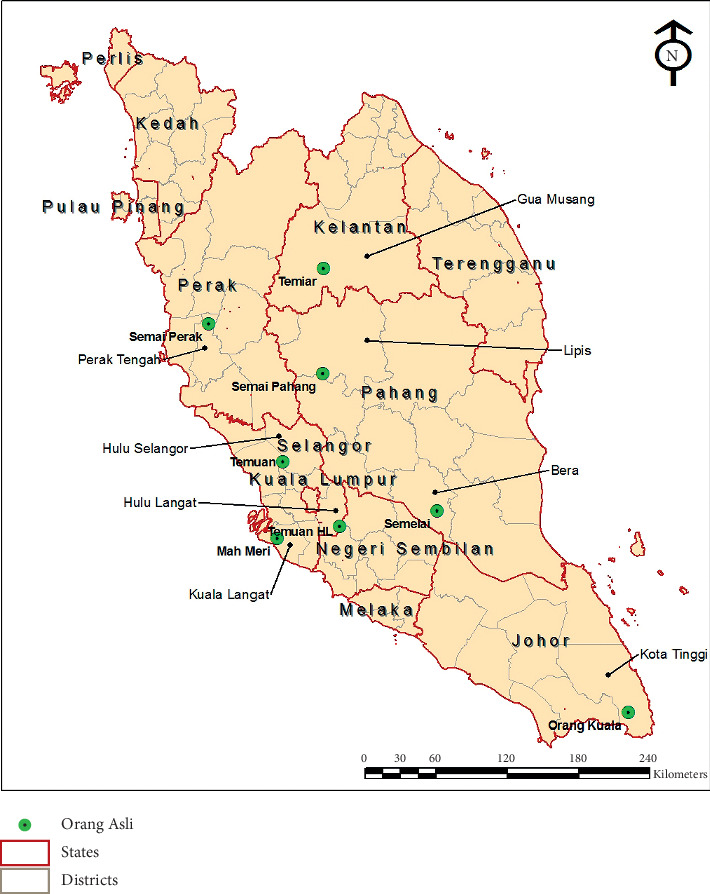
Map of Peninsular Malaysia showing the locations of the *Orang Asli* villages surveyed in the study. The red line shows the state division, while the gray line shows the division of districts in each state. The *Orang Asli* villages are indicated with green bubbles.

The serosurvey conducted was a cross-sectional study performed among OA populations residing in eight different OA villages in the forest or forest fringe areas of Peninsular Malaysia (Figure 1). The *Orang Asli* (OA) constituted about 0.6% of the Malaysian population and comprised of mainly 18 indigenous tribes (https://www.coac.org.my/). The sampling was performed between November 2007 and October 2010. No specific age was targeted as participation was on voluntary basis.

The villages in the present study were selected from the list of sites made available to the authors by JAKOA. The selection was based on the village's ease of access, its size (more than 100 individuals in the population), the villagers' receptivity to outsiders, and their nonnomadic lifestyle. As participation was on a voluntary basis, there were no inclusion or exclusion criteria for recruitment. A total of 716 participants were recruited; however, only 491 (68.6%) consented to blood withdrawal. The minimum number of sample size required was 246, calculated by EpiTools epidemiological calculator (epitools.ausvet.com.au) based on a 0.2 apparent prevalence, 0.5 estimated precision, 0.95 confidence level, and an estimated population size of 1,600.

The selected villages were the Sungai Perah village, the Sungai Bumbun village, the Gurney village, the Pos Iskandar village, the Hulu Langat village, the Kuala Betis village, the Pos Betau village, and the Sungai Layau village, located in different parts of Peninsular Malaysia (Table 1, Figure 1). The villages were located mostly in the forest fringe areas surrounded by rubber and oil palm plantations. In general, the villages had basic utility infrastructures such as water, electricity, and concrete houses. However, they were not fully utilized or evenly distributed as many could not afford the monthly utility bills. As such, the villagers depended highly on nearby rivers for daily water source. Villages such as Pos Iskandar, Kuala Betis, Pos Betau, and Sungai Layau underwent a resettlement program, which included improvement of nearby access roads. Although concrete houses were built, there were still many structures made from bamboo, wood, bricks, and Nipah palm trees. Each village had a population of more than 100 inhabitants. Most of the villagers were unskilled laborers employed at nearby construction sites, factories, vegetable farms, oil palm, and rubber plantations. The villagers also reared animals such as pigs, chickens, and ducks for food and kept monkeys, dogs, and cats as pets. These animals were mostly left to roam freely in the villages.

### 2.3. Structured Questionnaire Survey

The pretested questionnaire contained information on participant demographics (i.e., age, gender, and level of education attained) and socioeconomic status (i.e., occupation and household income; Table 1). The questionnaire was designed in the national language, Bahasa Malaysia, which was well understood by all of the participants. For those who were not fluent in the language, interpreters were provided by JAKOA. The questionnaire survey was performed by trained field investigators supervised by team supervisors. It was performed prior to blood withdrawal. Each answered questionnaire was given a unique identifier, and the same identifier was used for the blood samples. Completed forms were checked for accuracy, legibility, and completeness at the end of sampling day and verified by the team supervisors. The presence of a JAKOA official was required for all the visits.

### 2.4. Blood Collection

Approximately 3 ml of venous blood was drawn from each participant by trained medical assistants and nurses. The blood samples, in vacutainer blood tubes, were kept in chilled condition and transported to the Department of Parasitology, Faculty of Medicine, University of Malaya, after each study visit. The blood samples were immediately centrifuged at 500 *×*g for 10 min to obtain the serum. The serum was then stored at −20°C until further tests.

### 2.5. Dengue IgG Capture ELISA

The IgG capture enzyme-linked immunosorbent assay (ELISA) was performed using the Standard Diagnostics Dengue IgG Capture ELISA (SD, Korea; 11EK10) according to the recommended protocol. The absorbance was read at 450/620 nm using a Tecan Sunrise spectrophotometer (Mannedorf, Switzerland). The cutoff (CO) value was determined by adding 0.3 to the negative control's average absorbance value. An absorbance reading ≥CO value was considered positive for the presence of dengue-specific IgG.

### 2.6. Land Cover Analysis

The villages' location was determined using Google Earth 5.2.1 (https://www.google.com/Earth) as previously described [14]. Land cover assessment was made within a 2 km radius of the center of each village. Land cover features were divided into three categories: (1) water body, (2) built-up, and (3) vegetation. The water body was represented by rivers, streams, and ponds. Built-up consisted of residential, commercial, and industrial areas. They were identified based on the building design and location. For instance, industrial buildings would normally have a wider and bigger rooftop and located in the middle of a large clearing. A commercial area is usually found in a city center while a residential area is located at the city outskirts consisting of fairly homogenous structures. Categorization of the built-up was also assisted by Google Earth's denomination and by physical visits to the villages. Vegetation was represented mostly by forests and plantations. A plantation site was observed as patches of distinct homogenous pattern of greenery that consisted of oil palm and rubber plantations. These observations were also supported by the physical visits to the villages and the surrounding area. The estimation of land cover area was performed using GE-Path 1.4.4 (http://www.sgrillo.net/googleearth/gepath.htm) by creating a grid map overlay with the land cover map, enabling a quantitative assessment. One grid area was equivalent to 1 km^2^, and the study surveyed an area of 2 km radius from the center of the villages.

### 2.7. Remote Sensing Environmental-Derived Data

The Geographical Information System (GIS) was used to integrate survey data with remotely sensed satellite sensor environmental data. The data were typically provided as a raster file or in arrays of cells, in which each grid-cell, or pixel, had a certain value depending on how the image was captured and what it represented. There were three environmental data used in the present study: 1) the monthly average land surface temperature (LST); 2) the normalized difference vegetation index (NDVI); and 3) the digital elevation model (DEM). The land surface temperature data were obtained at 30 arcsec (∼1 km) resolution and downloaded from the WorldClim website (http://www.worldclim.org). Temperature records were produced from the global weather station for the period of 1950 to 2000 and were interpolated using a thin-plate smoothing spline algorithm [15].

The normalized difference vegetation index measures the vegetation density. It used a time series of a nominal 1 km spatial resolution from Moderate Resolution Imaging Spectroradiometer (MODIS) data that were downloaded from the NASA's Earth Observing System (EOS) data gateway (http://modis.gsfc.nasa.gov/data/dataprod/index.php). The normalized difference vegetation index was generated using a novel spline-based algorithm following the methods described by Scharlemann et al. [16]. The algorithm was tested on generated artificial data using randomly selected values of both amplitudes and phases, and it provided an accurate estimate of the input variables under all conditions. The algorithm was then applied to produce layers that captured the seasonality of the MODIS data. The digital elevation model information was generated from Radarsat data obtained from the Department of Survey and Mapping Malaysia.

A point estimate for each village was extracted for each environmental layer following a temporal Fourier analysis. They were transformed and analyzed using ESRI ArcGIS V9.3 software. A univariate (the Wald test) and multivariate (likelihood ratio test) logistic regression analysis with a stepwise procedure was performed to examine the relationship between remote sensing derived environmental variables and dengue seropositivity using STATA/IC 10.0 (StataCorp LP, College Station, Texas, USA).

### 2.8. Statistical Analyses

Statistical analyses were conducted using IBM SPSS 13.0 for Windows (Chicago, IL, USA). The initial data entry was crosschecked regularly to ensure that data were correctly and consistently entered. A percentage was used to describe descriptive data, such as the seroprevalence of dengue in the studied population according to the village, age, and gender. Univariate analysis was used to assess the potential associations between dengue seropositivity (the outcome of interest) and the sociodemographic characteristics. Only variables that were significantly associated in the univariate model were included in a logistic regression analysis using a backward elimination model. A significance level of *p* < 0.05 according to the odds ratios (OR) and a 95% confidence interval (95% CI) were used for all tests to indicate the strength of the association between dengue seropositivity and the respective variables.

## 3. Results

The present study recruited 491 individuals from eight different OA villages in Peninsular Malaysia (Figure 1). Their ages ranged from 1 to 82 years with a median age of 11 years old and a proportion of 1.2% of ≤4 years old, 2.2% of 5-6 years old, 70.1% of those aged 7–12 years, 3.5% of 13–17 years old, and 23.0% of those aged ≥18 years old.

Study participation was on a voluntary basis. The highest participation was obtained from the Pos Iskandar village (*n* = 109), followed by the Pos Betau village (*n* = 91), the Sungai Layau village (*n* = 88), and the Kuala Betis village (*n* = 77). There were 65 participations from the Sungai Perah village and 30 participations from the Hulu Langat village. The lowest participation was obtained from the Gurney (*n* = 16) and Sungai Bumbun village (*n* = 16; Table 1).

### 3.1. Dengue IgG Seroprevalence

Results from dengue IgG serological assays suggested that at least 17% (*n* = 83) of the studied population was positive for dengue IgG. The highest seropositivity was observed in the Sungai Perah population with 50% (*n* = 32) seropositivity (Table 1). This was followed by the Gurney and Sungai Bumbun villages with about 25% (*n* = 4) seropositivity. About 23.9% (*n* = 26) and 13.8% (*n* = 4) of the Pos Iskandar and Hulu Langat villagers were also dengue IgG positive. Less than 10% dengue IgG seropositivity was observed among volunteers from the Kuala Betis (9.1%), Pos Betau (4.4%), and Sungai Layau (2.3%) villages (Table 1).

### 3.2. Dengue IgG Seropositivity and Demographic and SocioEconomic Risk Factors

The demographic and socioeconomic variables that were analyzed consisted of the gender, age, level of education, occupational status, and monthly household income (Table 2). Univariate analysis of these risk factors identified females, those above 13 years old, with low education, and who are working participants as more likely to be seropositive for dengue (Table 2). Results showed that about 20.2% of female participants and 12.4% of male participants were dengue IgG seropositive (OR = 1.78; 95% CI = 1.08–2.95; *p*=0.023; Table 2). Dengue IgG seropositivity was also significantly different between those aged ≤12 years and ≥13 years old. About 10.6% of participants aged ≤12 years old and 34.6% of those aged ≥13 years old were positive for dengue IgG (OR = 4.45; 95% CI = 2.71–7.26; *p* < 0.001). Further analysis on the age groups showed that dengue IgG was found only in those aged more than 4 years old where those aged 5-6, 7–12, 13–17, and ≥18 showed 9.1%, 10.8%, 11.8%, and 38.1% seropositivity to dengue, respectively. There was a significant age-dependent increase of dengue seropositivity among the volunteers (*X*^2^ = 47.26; *p* < 0.001).

Results also showed that participants with no formal education, i.e., those who did not complete their six years of primary school, were significantly correlated with higher seropositivity for dengue in comparison to those with a formal education, i.e., those who had completed their six-year primary education (OR = 2.92; 95% CI = 2.00–4.27; *p* < 0.001; Table 2). In addition, those who had acquired jobs such as farming, hunting, as unskilled laborers, or more professional occupations such as teachers, nurses, and business entrepreneurs had significantly higher dengue seropositivity in comparison to those who did not possess a job (OR = 2.34; 95% CI = 1.54–3.56; *p* < 0.023). Analysis of the monthly income showed that those who earned a cumulative family income of less than MYR500 (USD150) were also more likely to have had dengue (OR = 2.23; 95% CI = 1.32–3.78; *p*=0.002).

Following a logistic regression analysis, only two groups of participants were significantly associated with dengue IgG seropositivity, those who earned a cumulative monthly income of less than MYR500 (∼USD150) and those whose age were more than 13 years old. Both groups were 2.2 and 4.4 times more likely to have had dengue in the past (95% CI = 1.86–6.60; *p* < 0.001; Table 2), respectively.

### 3.3. Seroprevalence of Dengue and Land Cover Analysis

In addition to investigating the potential association between dengue seroprevalence and the different demographic and socioeconomic variables, the study also explored the potential influence that land use or land cover has on the prevalence of dengue. The land cover variables that were analyzed were built-up consisting of residential, industrial, and commercial areas, vegetation consisting of forest and agriculture, and water bodies consisting of lake, river, and abandoned mine pools and ponds. The land cover analysis covered about 2 km radial distance from the center of the villages. Analysis of the surveyed areas showed that all of the villages were located near to a river stream. Rivers remained an important source of water for the OA despite the pipe water facility available at the villages as many could not afford the utility bills.

The Sungai Perah village, which had the highest dengue seropositivity (50%; Table 1) was the only village located near to an industrial area (Table 3), and both the Sungai Perah and Sungai Bumbun village were located near a commercial site. The highest built-up content was found around the Gurney village followed by the Sungai Perah village. In addition to the presence of a river stream, two other villages had a unique water body presence where the Sungai Bumbun village was located near to an abandoned tin mine pool, and a freshwater lake was spotted near the Pos Iskandar village. The freshwater lake, Tasik Bera, is the largest freshwater swamp in Peninsular Malaysia spanning about 35 km long and 20 km wide. The lake supports an array of animal and plant life and is an important source of livelihood for the Pos Iskandar villagers. The lake is often frequented by visitors and contributed significantly to the Pos Iskandar village's economy.

In addition to built-up and water bodies, the extent of vegetation was also estimated. The presence of vegetation was vast throughout the surveyed areas typical of equatorial rainforest consisting mainly of forested and agriculture areas. The highest content of forest was observed around the Kuala Betis village followed by the Pos Iskandar village. The least amount of vegetation and forest was observed around the Sungai Bumbun village followed by the Gurney village, where the latter also showed the highest content of built-up areas (Table 3).

The lowest dengue seroprevalence was observed among the Sungai Layau village residents. The village is located about 345 km southeast of Kuala Lumpur (Figure 1) and was mostly surrounded by an oil palm plantation. There was no significant difference in the land cover content between the Sungai Layau village and the Sungai Perah village (which exhibited the highest dengue prevalence) except for the built-up (Table 3). The Sungai Perah village had a much higher built-up (18.4%) in comparison to the Sungai Layau village (8.0%) with the presence of both industrial and commercial areas nearby, which the latter lacked. Although there was much less presence of built-up, the Sungai Layau village was more modern and developed in comparison to Sungai Perah and the other villages. The village has a health clinic and a primary and secondary school unlike the other villages. Many of the villagers completed their tertiary education and hold professional jobs.

Univariate analysis on the investigated variables showed that the different types of built-up and the presence of a lake or pond were significantly associated with dengue seroprevalence. However, multivariate analyses using logistic regression of these variables showed that only residential area (OR = 1.106; 95% CI = 1.041–1.175; *p* < 0.001) and lake (OR = 0.152; 95% CI = 0.067–0.348; *p* < 0.001) were significantly associated with higher seroprevalence of dengue (Table 4). Vegetation did not seem to correlate to dengue in the present study. The number of multilane roads, on the other hand, was associated with dengue prevalence (OR = 1.821; 95% CI = 1.471–2.252; *p* < 0.001), possibly indicating the role of movement or mobility in the spreading of the disease.

### 3.4. Remote Sensing Environmental Data

Three environmental data were assessed in the present study: land surface temperature (LST), land elevation using the digital elevation model (DEM), and the level of vegetation represented by the normalized difference vegetation index (NDVI). A univariate analysis was performed followed by the multivariable logistic regression model in order to determine the potential influence that physical environmental factors have over disease prevalence and possibly spread. Results suggested that LST exceeding 40°C (OR = 1.107, 95% CI = 0.86–1.40, *p*=0.05) and elevation less than 50 meters above sea level (OR = 2.210, 95% CI = 1.51–2.63, *p*=0.04) were significantly associated with >20% dengue IgG seropositivity such as in Sungai Perah, Gurney, Sungai Bumbun, and Pos Iskandar villages. Similar to the previous analyses, NDVI showed no significant association with dengue prevalence (OR = 0.976, 95% CI = 0.94–1.01, *p*=0.13), suggesting its minimal role in possibly influencing dengue transmission (Table 3).

## 4. Discussion

Activities such as the opening of oil palm and rubber plantations, timber extraction, and eco-tourism have resulted in substantial land surface changes in many tropical and subtropical regions of the world. To ease travelling and the transport of forest resources, workers, and tourists, highways and multilane roads were built, contributing to an increase in population movement to and from the forest fringe areas. Although the increase in mobility helped to boost economic activities, it also may inadvertently increase the chances for transmission of infectious diseases. Dengue, a mosquito-borne disease and hyperendemic in Malaysia, remained a serious public health threat. Although the disease is mandated as a notifiable disease within 24 hour of detection, it is most likely still under reported especially in population where minimal health services are available, such as among the OA or those living in the forest fringe areas. Only few studies have been undertaken to investigate the prevalence of dengue in these populations [10–13], and none of them attempted to determine potential factors that could influence disease transmission and prevalence. The earlier studies conducted in 1956 [10] and 1958 [11] reported high prevalence of dengue (>90%) among rural ethnic Malays and OA in Pahang and an 80% prevalence among the forest fringe populations [12]. The prevalence increased to 91.6% in 2011 in rural areas [14] but varied significantly (24% prevalence) among the forest fringe populations of East Malaysia in the year 2006 [13]. Following the report in 1986 [12], the present study attempted to determine the prevalence of dengue among the forest fringe populations in Peninsular Malaysia and associate it with demographic and socioeconomic factors in addition to land cover and aspects of environment such as LST, land elevation, and vegetation.

The present study observed a low prevalence of dengue (17%) among the forest fringe populations in comparison to those reported earlier. An even lower prevalence of dengue (4.9%) was reported more recently where the study also showed significantly higher presence of antibodies against Japanese encephalitis (48.4%) among the OA and some presence of IgG antibodies against Zika (13.2%) [17]. This serosurvey suggested that dengue prevalence had decreased over time among those living in forest fringe areas and that it is significantly different from that of the rural areas. Increasing dengue trend in the rural area was estimated to reach as high as that in the urban areas if not higher [18, 19]. Although the study by Schmidt et al. showed that the lack of piped water supply contributed to higher dengue prevalence in the rural areas of Vietnam, the same was not observed among the villages surveyed in the present study [19].

Despite the overall low dengue seroprevalence in the present study, there was a significant difference of dengue exposure across the surveyed villages, ranging from 2% in the Sungai Layau village to 50% in the Sungai Perah village. Upon comparison of these two villages, no particular differences were observed, except for the presence of an industrial area near Sungai Perah. Industrial area, however, was not shown to be a significant contributor to dengue seroprevalence in contrast to the presence and size of residential areas. Based on the assumption that residential area reflected the number of families or individuals present in each village, this could indicate the importance of density or crowding in the transmission and prevalence of dengue in the forest fringe areas [20].

Our study also showed mobility as a cofactor that contributed to high seropositivity of dengue. This was reflected by the higher dengue seropositivity detected among the Pos Iskandar participants in comparison to the Kuala Lipis participants where the Pos Iskandar village was located near to Tasik Bera, the largest natural freshwater lake in Malaysia. The lake area is inhabited by the OA from the Semelai tribe, who is also known as the lake people and is frequently visited by eco-tourists. Due to the traffic and population movement to Tasik Bera, the risk of bringing in DENV from either asymptomatic or viremic individuals increased especially with ample presence of vector mosquitoes in the surrounding areas. The role of mobility in the dispersion and prevalence of dengue was made more evident when the Pos Iskandar village was compared to the Kuala Lipis village where both were located near to only one multilane road, suggesting similar accessibility; however, only 5.3% of the Kuala Lipis participants were exposed to dengue in comparison to 23.9% of the Pos Iskandar participants. The suggestion that mobility plays an important role in the spread of diseases is consistent with other earlier studies [21, 22].

In addition to population crowding and mobility, previous studies have shown that demographic and socioeconomic attributes contributed significantly to the prevalence of dengue. Lower socioeconomic status and age has been consistently associated with dengue in a number of studies [20, 23–25]. In impoverished areas such as in Recife, Brazil, dengue prevalence was as high as 59% among children ≤5 years old [20]. Similarly, the present study showed that dengue was inversely associated with wealth and socioeconomic status even among the forest fringe populations of OA and that increasing age was a significant variable associated with dengue. There was a display of age-dependent increase of prevalence in the present study, with those above 18 years old displaying the highest seropositivity to dengue (38.1%) and those earning the very bare minimum of ∼USD150 were more likely to be exposed to the disease. In our study, better accessibility to healthcare and education could be important factors in reducing dengue transmission. Empowering the community with disease knowledge and prevention practices, hence, would likely assist in curbing the spread of dengue [26].

Environmental variables such as LST and elevation were also found to contribute to the prevalence of dengue. Land surface temperature (LST) and elevation played a role by possibly influencing the vectorial capacity of dengue vector, the *Aedes* sp. Mosquitoes [27–30] where the abundance of *A. aegypti* has been shown to reduce significantly at elevation higher than 1,700 m [28, 29]. Higher LST was also associated with high occurrence of severe dengue in four provinces in Thailand [30]. These two environmental factors could be included in a simple predictive algorithm to determine dengue expansion, just as they have been used in the development of a K-map model to visualize dengue hot spot areas [31]. Vegetation, however, was not shown as a significant contributor to the prevalence of dengue in the present study despite the perception of higher abundance of mosquitoes in highly vegetated areas and the previous display of association between greenery and the number of dengue cases [32].

Despite the presented results, the present study would have benefit from the use of a more sophisticated land survey methods such as an unmanned aerial vehicle where higher resolution satellite images could be obtained. Inclusion of climatic variables such as air temperature and rainfall should be considered in future studies, as is mosquito population and density. In addition, future studies should also address issues of dengue cross-reactivity with other arboviruses such as Zika and Japanese encephalitis in serological assays [17]. Since the study was performed approximately 10 years ago, it is possible that much societal and climate changes have occurred over the years, which could have affected the present dengue serological status among the OA. This, however, remained to be assessed. Further studies, hence, are needed to ascertain the degree of influence that the examined variables have on dengue transmission and prevalence in these forest fringe OA populations.

## 5. Conclusion

The present study highlighted the prevalence of dengue among the underserved and economically marginalized OA population of Malaysia. Variables such as population mobility, household density, age, and lower socioeconomic status are among the risk factors for dengue identified in the study. In addition, environmental factors consisting of LST and elevation appeared to also influence the prevalence of dengue. These factors, however, are not solely exclusive to populations living in forest fringe areas but could also be true in other underserved or economically marginalized population. Better access to healthcare and empowerment with disease knowledge is recommended to ensure better success of preventive measures against dengue in these populations.

## Figures and Tables

**Table 1:** Location of the surveyed villages, their accessibility, and information on dengue prevalence.

Surveyed villages	Location		*N* ^*∗*^	Dengue serology positive		Nearby multilane roads
	Longitude (°E)	Latitude (°N)		*N*	%	
Sungai Perah	100° 54 72″	4° 24″ 288″	65 (43%)	32	50.0	4
Gurney	101° 24″ 144″	3° 24″ 108″	16 (11%)	4	25.0	4
Sungai Bumbun	101° 24″ 72″	2° 48″ 180″	16 (11%)	4	25.0	3
Pos Iskandar	102° 36″ 180″	3° 0″ 216″	109 (73%)	26	23.9	1
Hulu Langat	101° 54″ 36″	2° 54″ 144″	29 (19%)	4	13.8	2
Kuala Betis	101° 42″ 324″	4° 54″ 0″	77 (51%)	7	9.1	2
Pos Betau	101° 46″ 48″	4° 6″ 0″	91 (61%)	4	4.4	1
Sungai Layau	104° 6″ 0″	1° 30″ 108″	88 (59%)	2	2.3	2

^*∗*^The percentage of participation was estimated based on an average population of 150 per village.

**Table 2:** Analysis of potential risk factors associated with dengue seroprevalence among the *Orang Asli* communities in Peninsular Malaysia (*N* = 491).

Variables	*N*	Dengue serology positive		OR (95% CI)	*p* value
		*N*	%	
*Gender*					
Female	282	57	20.2	1.78 (1.08–2.95)	0.023
Male	209	26	12.4	1	

*Age (years)* ^*∗*^					
≥13 years	130	45	34.6	4.43 (2.71–7.26)	<0.001
≤12 years	361	38	10.6	1	

*Level of education*					
No formal education	123	41	33.3	2.92 (2.00–4.27)	<0.001
Formal education	368	42	11.4	1	

*Occupational status*					
Working	62	21	33.9	2.34 (1.54–3.56)	<0.001
Not working	429	62	14.5	1	

*Household income (RM/month)* ^*∗*^					
<RM 500	329	68	20.7	2.23 (1.32–3.78)	<0.001
>RM 500	162	15	9.3	1	

^*∗*^Variables that were significantly associated with dengue prevalence following a; multivariate analysis. A significant association is indicated by *p* < 0.05. OR value of 1 is the reference group. *N*: number examined; No: number positive; %: percentage; significant association (*p* < 0.05); reference group marked as OR = 1.

**Table 3:** Percentage of coverage for the different types of land cover surrounding 2 km radial distance from the center of each surveyed village.

Surveyed villages	Landcover attributes (% of coverage)								
	Residential	Industrial	Commercial	Lake	River	Mine pool	Pond	Forest	Agriculture
Sungai Perah	14.6	2.3	1.5	—	13.8	—	—	29.3	38.5
Gurney	29.6	—^*∗*^	—	—	12.6	—	5.9	14.9	37.0
Sungai Bumbun	11.5	—	3.1	—	4.6	50.0	15.4	11.6	3.8
Pos Iskandar	3.8	—	—	30.8	5.4	—	—	46.2	13.8
Hulu LANGAT	14.2	—	—	—	2.5	—	—	29.2	54.1
Kuala Betis	7.7	—	—	—	2.3	—	—	66.9	23.1
POS BETAU	5.4	—	—	—	2.3	—	—	38.5	53.8
Sungai Layau	8.0	—	—	—	24.8	—	12.0	23.2	32.0

^*∗*^Not available.

**Table 4:** Multivariate analysis of potential land cover risk factors associated with dengue seroprevalence among the *Orang Asli* communities living in the forest fringe areas of Peninsular Malaysia.

Land cover variables	*p* value	Exp (*B*)	95% C.I. for exp(B)	
			Lower	Upper
Residential	<0.001	1.106	1.041	1.175
Industrial	0.171	0.354	0.080	1.564
Commercial	0.061	1.884	0.971	3.657
Lake	<0.001	0.152	0.067	0.348
Pond	0.146	0.906	0.792	1.035
Multi-lane roads	<0.001	1.821	1.471	2.252
Land surface temperature (LST)	0.050	1.107	0.860	1.400
Land elevation (DEM)	0.040	2.210	1.510	2.630
Normalized difference vegetation index (NDVI)	0.130	0.976	0.940	1.010

Significant association is indicated by *p* < 0.005.

## Data Availability

The data used to support the findings of this study are included within the article.
